# C-Terminal Binding Proteins Promote Neurogenesis and Oligodendrogenesis in the Subventricular Zone

**DOI:** 10.3389/fcell.2020.584220

**Published:** 2021-01-06

**Authors:** Catarina Serra-Almeida, Cláudia Saraiva, Marta Esteves, Raquel Ferreira, Tiago Santos, Ana Clara Cristóvão, Liliana Bernardino

**Affiliations:** ^1^Faculty of Health Sciences, Health Sciences Research Centre (CICS-UBI), University of Beira Interior, Covilhã, Portugal; ^2^NeuroSoV, UBImedical, University of Beira Interior, Covilhã, Portugal

**Keywords:** neural stem cells, subventricular zone, C-terminal binding proteins, neurogenesis, oligodendrogenesis

## Abstract

C-terminal binding proteins (CtBPs) are transcriptional modulators that can regulate gene expression through the recruitment of a corepressor complex composed of chromatin-modifying enzymes and transcriptional factors. In the brain, CtBPs have been described as regulators of cell proliferation, differentiation, and survival. Nevertheless, the role of CtBPs on postnatal neural stem cells (NSCs) fate is not known yet. Herein, we evaluate the expression and functions of CtBPs in postnatal NSCs from the subventricular zone (SVZ). We found that CtBPs were expressed in immature/progenitor cells, neurons and glial cells in the SVZ niche. Using the CtBPs modulator 4-methylthio 2-oxobutyric acid (MTOB), our results showed that 1 mM of MTOB induced cell death, while 5, 25, and 50 μM increased the number of proliferating neuroblasts, mature neurons, and oligodendrocytes. Interestingly, it also increased the dendritic complexity of immature neurons. Altogether, our results highlight CtBPs putative application for brain regenerative applications.

## Introduction

Neural stem cells (NSCs) are multipotent cells that can proliferate through long-term self-renewal and differentiate into neurons and glial cells (Pino et al., 2017). Therefore, the modulation of NSCs behavior represents a promising approach for the development of potential therapeutic strategies for brain repair. These cells persist in the adult mammalian brain in two main neurogenic niches, the subventricular zone (SVZ) along the walls of the lateral ventricles, and in the subgranular zone (SGZ) of the hippocampus (Garcia et al., 2004). SVZ-derived NSCs can differentiate into interneurons in the olfactory bulb and they are also a source of oligodendrocytic progenitor cells that generate myelinating oligodendrocytes (Kriegstein and Alvarez-Buylla, 2009; Obernier and Alvarez-Buylla, 2019). Several intrinsic factors, such as transcriptional factors and epigenetic modifications may impact on NSC fate (Ming and Song, 2011).

Among these factors, C-terminal binding proteins (CtBPs) are potential candidates as they were also initially linked to tumorigenesis. CtBPs act mainly by repressing gene expression through the recruitment of a corepressor complex, that includes chromatin-modifying enzymes, to DNA-binding transcriptional factors (Chinnadurai, 2002). In mammals, CtBPs are encoded by two genes, CtBP1 and CtBP2, that originate different isoforms by alternative splicing (Verger et al., 2006). CtBP1 and CtBP2 contain the PXDLS- and RRT-binding motifs, crucial for the recruitment of the corepressor complex (Kuppuswamy et al., 2008). Nonetheless, only CtBP2 long isoforms display a nuclear localization signal that contributes to their nuclear localization (Verger et al., 2006). Besides their role as translational corepressors in the nucleus, CtBPs also exert cytosolic functions, such as membrane fission (Valente et al., 2013). Although CtBPs are mainly described as transcriptional corepressors, growing evidence suggest that CtBPs may also act as coactivators (Fang et al., 2006; Itoh et al., 2013).

C-terminal binding proteins are highly expressed in the nervous system during embryonic development (Furusawa et al., 1999). The genetic deletion of CtBP1 and CtBP2 results in severe developmental defects and embryonic lethality (Hildebrand and Soriano, 2002). Moreover, CtBPs have been shown to regulate neuronal differentiation in the neural tube (Xie et al., 2011; Dias et al., 2014). Recent evidence point out that CtBP2 is widely expressed in neural stem and progenitor cells and neurons in the developing cortex, being required for the proliferation and migration of these cells as well as for proper neuronal differentiation and maturation (Karaca et al., 2020). In addition to neuronal development, CtBPs may regulate neuronal survival and function (Hübler et al., 2012). Nevertheless, the role of CtBP1 and CtBP2 in NSCs in the postnatal and adult brain is not yet known. Thus, this work aims to explore the expression and the functions of CtBPs in NSCs from the SVZ. First, we have found that both CtBP1 and CtBP2 are expressed in immature/progenitor cells, neurons and glial cells present in the SVZ niche. Then, by modulating CtBP activity with its substrate 4-methylthio 2-oxobutyric acid (MTOB), we have found that low concentrations induced differentiation of NSCs into fully mature neurons and myelinating oligodendrocytes. Thus, the fine-tuned modulation of CtBP activity may reveal a novel target for brain regenerative approaches.

## Materials and Methods

### *In vivo* Studies

All animal experiments were conducted in agreement with protocols approved by the national ethical requirements for animal research, and in accordance with the European Community guidelines (2010/63/EU). Adult wild-type C57BL/6J male mice (8- to 10-week-old) were used for the *in vivo* experiments. Mice were kept in appropriate cages, under 12 h light/dark cycle in room temperature (RT; 22°C) and *ad libitum* access to food and water.

### Tissue Collection

Mice were deeply anesthetized with an intraperitoneal injection of a mixture of ketamine (90 mg/kg of mouse weight; Imalgene 1000, Merial, France) and xylazine (10 mg/kg of mouse weight; Rompun 2%, Bayer, Germany) and euthanized by transcardial perfusion with 0.9% NaCl followed by perfusion with 4% paraformaldehyde (PFA; Sigma-Aldrich). Brains were surgically removed and post-fixed overnight with 4% PFA, followed by immersion in a 30% sucrose solution, until sunk. Then, brains were cryopreserved, and 40 μm-thick coronal sections of the SVZ/striatum were collected using a freezing cryostat-microtome (Leica CM 3050S, Leica Microsystems, Nussloch, Germany). Slices were kept at −20°C in an antifreeze solution made in 0.1 M phosphate-buffered saline (PBS) containing 30% glycerol and 30% ethylene glycol, until immunohistochemistry stainings.

### Immunohistochemistry

Brain slices were rinsed with PBS, permeabilized and blocked to prevent unspecific bindings with 2% horse serum (Life Technologies) and 0.3% Triton X-100 in PBS, for 2 h at RT. Then, slices were incubated with the primary antibodies prepared in the blocking/permeabilization solution for 48 h at 4°C. Primary antibodies used are listed in the Supplementary Table 1. Brain slices were rinsed with PBS and incubated with the respective secondary antibodies (Supplementary Table 1) together with Hoechst-33342 (1:1000; Life Technologies), for nuclear staining, in PBS containing 0.3% Triton X-100, for 2 h at RT. Lastly, slices were rinsed with PBS and mounted in Fluoroshield Mounting Medium (Abcam Plc.). All these steps were performed using an orbital shaker. Representative fluorescent images were acquired using an AxioObserver LSM 710 confocal microscope (Carl Zeiss) with a total magnification of 400x.

### Primary SVZ Cell Cultures and MTOB Experimental Treatments

Subventricular zone cells were isolated from 1- to 3-day-old C57BL/6J mice, as described by us (Agasse et al., 2008). Animals were sacrificed by decapitation and brains were removed and placed into Hanks Balanced Salt Solution (HBSS; Gibco) supplemented with 100 U/mL penicillin and 100 μg/mL streptomycin (all from Life Technologies). SVZ fragments were dissected from 450 μm-thick coronal brain sections using a McIlwain tissue chopper. The fragments of SVZ were placed into HBSS supplemented with 100 U/mL penicillin and 100 μg/mL streptomycin and digested in 0.025% trypsin and 0.265 mM EDTA (all from Life Technologies), followed by mechanical dissociation. Cell suspension was diluted in serum-free medium composed by Dulbecco’s Modified Eagle Medium/Nutrient Mixture F-12 (DMEM/F-12) (DMEM/F-12 + GlutaMAX^TM^-I; Gibco) supplemented with 100 U/mL penicillin, 100 μg/mL streptomycin, 1% B27 supplement, 10 ng/mL epidermal growth factor (EGF) and 5 ng/mL basic fibroblast growth factor-2 (FGF-2) (all from Life Technologies). Single cells were plated on uncoated Petri dishes (Corning Life Science) and allowed to develop in an incubator with 5% CO_2_ and 95% atmospheric air, at 37°C.

Six-day-old neurospheres were seeded onto poly-D-lysine (PDL; 0.1 mg/mL; Sigma-Aldrich)-coated glass coverslips in 24-well plates, in the presence of DMEM/F-12 devoid of growth factors (EGF, FGF-2), for 2 days. To investigate the effects of the substrate-based inhibitor of CtBPs, MTOB (Sigma-Aldrich), SVZ cells were treated with 5 μM, 25 μM, 50 μM, 100 μM, 1 mM, and 2.5 mM of MTOB diluted in DMEM/F-12 devoid of growth factors. Controls were included in all experiments. Then, SVZ cells were placed in an incubator with 5% CO_2_ and 95% atmospheric air, at 37°C, for several time points until fixation.

### Propidium Iodide Incorporation

Propidium iodide (PI; 5 μg/mL; Sigma-Aldrich) was used to quantify the numbers of necrotic and late apoptotic cells (Santos et al., 2017). Ten minutes before the end of the 48 h treatments, PI was added to cell medium at 37°C. Cells were fixed using 10% formalin solution for 15 min, at RT, and rinsed with PBS. Hereafter, cell nuclei were stained with Hoechst-33342 (1:500) and mounted in Fluoroshield Mounting Medium. Five random microscopic fields were acquired per replicate using an AxioImager microscope (Carl Zeiss) with a total magnification of 400×. The number of PI-positive (PI^+^) cells and fragmented/condensed nuclei was obtained using the ImageJ software (NIH, Bethesda, MD, United States).

### Immunocytochemistry Analysis

To investigate the cellular expression of CtBP1 and CtBP2 in the SVZ, cells were fixed 48 h and 7 days after neurospheres have been seeded. To assess the effects of CtBPs in NSC fate, cells were fixed at 48 h (cell commitment and dendritic morphology) or 7 days (cell differentiation, dendritic complexity, and myelinization of neurites) after MTOB treatments.

Subventricular zone cells were fixed using 10% formalin solution, for 15 min, at RT, and rinsed with PBS. The permeabilization and blocking step was then performed with 0.5% Triton X-100 and 3% bovine serum albumin (BSA), for 30 min (cytoplasmic stainings) or with 0.5% Triton X-100 and 6% BSA for 1 h (nuclear stainings) at RT. Cells were incubated overnight with the primary antibodies prepared in 0.3% BSA and 0.1% Triton X-100 diluted in PBS at 4°C. Primary antibodies are listed in the Supplementary Table 1. Thereafter, cells were rinsed with PBS and incubated with the secondary antibodies (Supplementary Table 1) together with Hoechst-33342 (1:500) nuclear staining in PBS for 1 h at RT. Lastly, cells were rinsed with PBS and mounted in Fluoroshield Mounting Medium. Immunocytochemistry analyzes were performed at the border of seeded neurospheres where SVZ cells formed a pseudo-monolayer.

For the evaluation of CtBPs expression, representative images were acquired using a confocal microscope (LSM 710; Carl Zeiss) under a 40× oil immersion objective.

For the assessment of the effects of CtBPs in cell commitment and differentiation, five random microscopic fields were acquired per replicate using an AxioObserver LSM 710 confocal under a 40× oil immersion objective. The number of cells was obtained using the ImageJ software.

For the quantification of TUJ1-positive (TUJ1^+^) neurites that were ensheathed by MBP-positive (MBP^+^) oligodendrocytes, fluorescence z-stack images of five random microscopic fields were acquired per replicate using an AxioObserver LSM 710 confocal microscope under a 40× oil immersion objective. The Colocalization Analysis/Colocalization Highlighter ImageJ plugin was used to highlight the regions where TUJ1^+^ neurites and MBP^+^ oligodendrocytes colocalize. Then, the quantitative analysis of the intensity of the overlay regions between TUJ1^+^ and MBP^+^ stainings was performed using the ImageJ Software.

For dendritic morphology analysis, fluorescence immunostaining z-stack images of DCX-positive (DCX^+^) cells were obtained using an AxioObserver LSM 710 confocal microscope under a 40× oil immersion objective and a ×0.7 digital zoom. For this analysis, a total of 30 DCX^+^ cells were assessed based on their distance to the closer neurosphere. Dendritic morphology of DCX^+^ cells was analyzed in the Fiji software (NIH, Bethesda, MD, United States) using the Simple Neurite Tracer plugin (Longair et al., 2011). This semi-automatic approach allowed us to perform Sholl analysis that characterizes neuronal arbors based on the quantification of the number of intersections between dendrites and the circles with a radius increment of 5 μm as well as to measure neuron length and volume.

### Statistical Analysis

Each condition of the immunocytochemistry experiments was analyzed from two coverslips per SVZ culture and counting was made in five random fields per replicate. Each coverslip was considered a replicate of the same experiment (*n*). About 150–200 cells were counted per microscopic field. Data are expressed as mean ± standard error of the mean (SEM), presented as either percentage of values obtained in control condition or absolute values. Statistical analysis was performed using one-way ANOVA, followed by Dunnett’s Multiple Comparison Test for comparison with the control condition. For dendritic morphology analysis, statistical analysis was performed using a two-way ANOVA, followed by Sidak’s Multiple Comparison Test or unpaired Student’s *t*-tests. Values of *p* < 0.05 were considered significant. All statistical analysis was made using GraphPad Prism 8 (GraphPad Software, San Diego, CA, United States).

## Results

### CtBPs Are Expressed in Immature/Progenitor and Differentiated Cells in the SVZ

**FIGURE 1:**
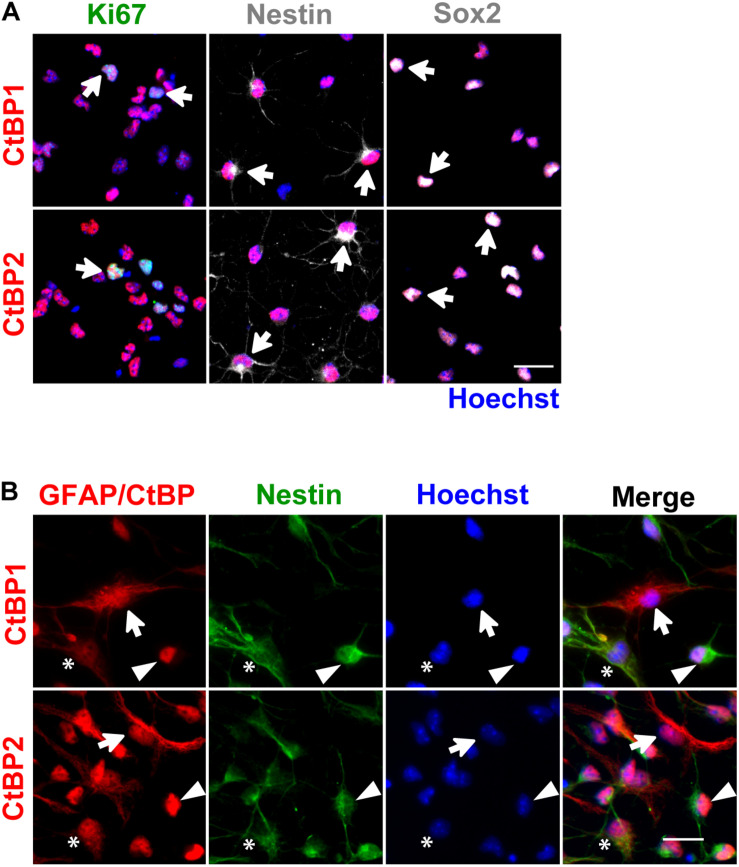
CtBP1 and CtBP2 are expressed in immature/progenitor cells. **(A)** Representative confocal images of the expression of CtBPs in proliferating (Ki67) and immature cells (Nestin, Sox2), evaluated 48 h after 6-day-old neurospheres have been seeded in coverslips. **(B)** Representative images of the staining against CtBPs, GFAP, and Nestin, evaluated 48 h after neurospheres have been seeded in coverslips. Both CtBP1 and CtBP2 are expressed in immature Nestin^+^GFAP^+^ (asterisks) and Nestin^+^GFAP^−^ (arrowheads) cells and astroglial Nestin^−^GFAP^+^ (arrows) cells. Nuclei are shown in blue (Hoechst staining). Scale bar: 20 μm.

**FIGURE 2:**
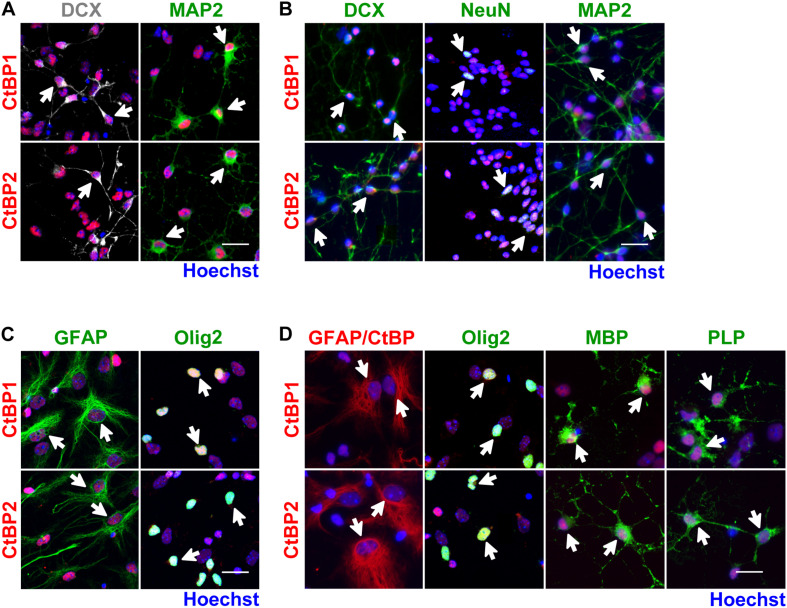
CtBP1 and CtBP2 are expressed in neurons and glia derived from SVZ NSCs. **(A,B)** Representative confocal images of the expression of CtBPs in neuroblasts (DCX) and neurons (MAP2 or NeuN), evaluated **(A)** 48 h and **(B)** 7 days after 6-day-old neurospheres have been seeded in coverslips. **(C,D)** Representative confocal images of the expression of CtBPs in **(C)** astrocyte-like cells (GFAP) and in oligodendrocytes/type C cells (Olig2), evaluated 48 h after 6-day-old neurospheres have been seeded in coverslips and in **(D)** astrocytes (GFAP) and oligodendrocytes (Olig2, MBP, and PLP), evaluated 7 days after the seeding of neurospheres. Nuclei are shown in blue (Hoechst staining). White arrows highlight cells with double staining. Scale bar: 20 μm.

*In vitro*, SVZ NSCs are initially grown in proliferative conditions, forming neurospheres that are enriched in progenitors and immature cells. Then, under differentiation conditions, neurospheres give rise to several cell phenotypes, mimicking several key features of the *in vivo* SVZ niche (Lim and Alvarez-Buylla, 2016). Herein, SVZ cells were cultured under differentiation conditions for 48 h and for 7 days, followed by immunocytochemistry against CtBP1 or CtBP2 together with several markers for proliferating (Ki67) and immature cells (Nestin and Sox2), neuroblasts (DCX), neurons (NeuN and MAP2), astrocytes-like cells (GFAP), oligodendrocytes/type C cells (Olig2) and mature oligodendrocytes (MBP and PLP). CtBP1 and CtBP2 are expressed in Ki67^+^, Nestin^+^, and Sox2^+^ immature cells (Figure 1A), after 48 h of differentiation, showing a nuclear localization. Specifically, CtBPs are expressed both in immature Nestin^+^/GFAP^+^ and Nestin^+^/GFAP^–^ cells and in astroglial Nestin^–^/GFAP^+^ cells (Figure 1B). Moreover, CtBPs are also expressed in neuronal (Figures 2A,B) and glial cells (Figures 2C,D), both at short (48 h) and long-term (7 days) differentiation protocols, showing a nuclear localization. Further analysis revealed that both CtBP1 and CtBP2 are expressed in more than 90% of the total cells at 48 h and 7 days of the differentiation protocol (data not shown). Additionally, CtBPs protein levels were similar between cultures grown under differentiation or proliferative conditions (Supplementary Figure 1).

**FIGURE 3:**
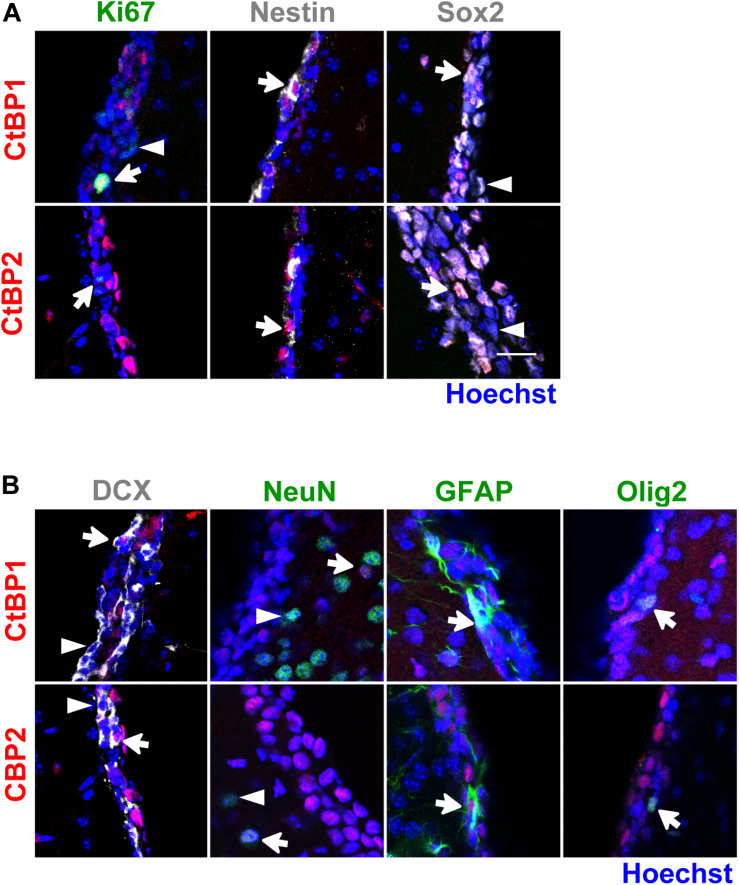
CtBP1 and CtBP2 are expressed in immature/progenitor cells, neuroblasts and glial cells in the SVZ neurogenic niche *in vivo*. **(A,B)** Representative confocal images of the expression of CtBPs in **(A)** proliferating (Ki67) and immature cells (Nestin, Sox2), and in **(B)** neuroblasts (DCX), mature neurons (NeuN), astrocytes (GFAP), and oligodendrocytes (Olig2), evaluated in the SVZ niche of wild-type C57BL/6J adult mice. Nuclei are shown in blue. White arrows highlight cells with double staining whereas arrowheads highlight cells that do not express CtBP1 or CtBP2. Scale bar: 20 μm.

In the SVZ niche *in vivo*, both CtBPs were expressed in the nuclei of immature/progenitor (Ki67^+^, Nestin^+^, Sox2^+^; Figure 3A) and glial cells (GFAP^+^, Olig2^+^; Figure 3B). In neuroblasts (DCX^+^), CtBP2 was found in the nuclei of some of these cells whereas CtBP1 was found both in the nuclei (Figure 3B) and in the cytoplasm (Supplementary Figure 2). Interestingly, CtBPs were expressed only in subpopulations of the cells, in contrast with *in vitro* findings. As expected, NeuN^+^ cells were not found in the SVZ niche but both CtBPs localized in the nucleus of some NeuN^+^ cells in the striatum (Figure 3B).

### High Concentrations of MTOB Trigger Cell Death in SVZ Cells

Then, to analyze the effects of CtBPs in SVZ cell survival, we have used MTOB, an α-ketoacid substrate-based inhibitor of CtBPs that shows a biphasic saturation curve. At low concentrations, MTOB acts as a substrate, whereas at high concentrations, it acts as a dehydrogenase inhibitor by preventing the NADH-dependent dimerization of CtBPs and suppressing their downstream activity (Achouri et al., 2007). Also, it displays a high affinity for CtBPs due to their interaction at the active site tryptophan which is unique among other dehydrogenases (Hilbert et al., 2014).

**FIGURE 4:**
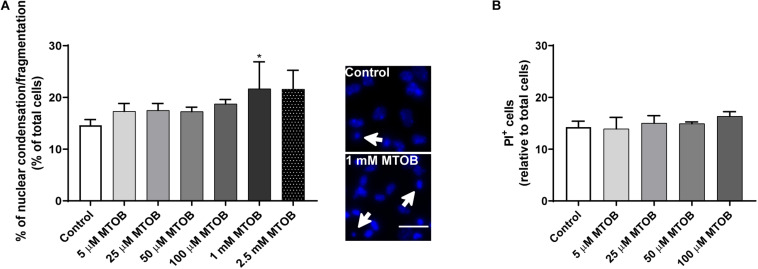
High concentrations of MTOB induce SVZ cell death. **(A,B)** Percentage of **(A)** condensed/fragmented nuclei and **(B)** PI^+^ cells assessed 48 h after MTOB treatments. Data are expressed as mean ± SEM. **(A)** *n* = 2-8 and **(B)** *n* = 4 independent experiments. *p < 0.05 when compared to control, using one-way ANOVA, followed by Dunnett’s multiple comparison test. Representative images of Hoechst-labeled nuclei are shown next to the graph, with white arrows highlighting condensed/fragmented nuclei. Scale bar: 20 μm.

Subventricular zone cells were treated with increasing concentrations of MTOB (5 μM, 25 μM, 50 μM, 100 μM, 1 mM and 2.5 mM) for 48 h. Then, cell death was evaluated by nuclear condensation/fragmentation (Figure 4A) and PI incorporation (Figure 4B) analysis. High concentrations of MTOB, namely 1 mM, increased significantly the number of condensed/fragmented nuclei [Figure 4A; control: 14.63 ± 1.1%; 1 mM: 21.68 ± 5.2% (*p* = 0.0497)]. Based on these results, we then used non-toxic MTOB concentrations only, up to 100 μM, to quantify the number of late apoptotic and necrotic cells by PI staining. MTOB did not induce a significant increase in the number of PI^+^ cells when used until 100 μM (Figure 4B). Due to the toxic effect of the high concentrations of MTOB, only the concentrations up to 100 μM of MTOB were chosen for the following experiments.

### CtBP Modulation Promotes Neuronal Differentiation

**FIGURE 5:**
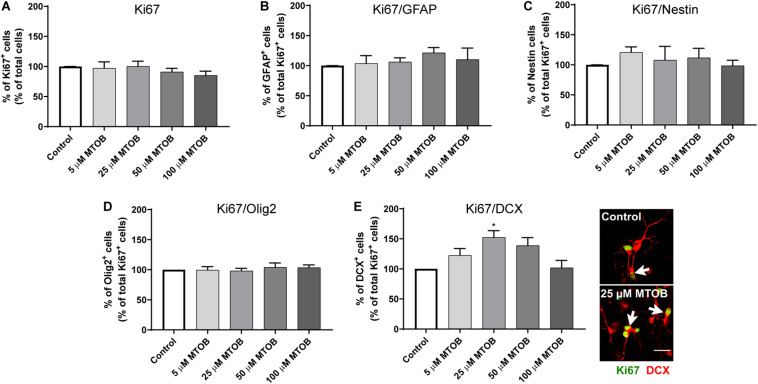
CtBP modulation increases neuroblasts proliferation. **(A)** Bar graph depicts the percentage of Ki67^+^ cells, evaluated 48 h after MTOB treatments. **(B–E)** Bar graphs depict the percentages of **(B)** GFAP^+^, **(C)** Nestin^+^, **(D)** Olig2^+^, or **(E)** DCX^+^ cells co-stained with Ki67. Data are expressed as percentage of control ± SEM. **(A)** *n* = 7–9, **(B)** *n* = 2–3, **(C)** *n* = 5–6, **(D)** *n* = 3, and **(E)** *n* = 3–4 independent experiments. Control was set to 100%. *p < 0.05 when compared to control, using one-way ANOVA, followed by Dunnett’s multiple comparison test. Representative confocal images of Ki67/DCX immunostaining are shown next to the graph, with white arrows highlighting cells with double staining. Scale bar: 20 μm.

**FIGURE 6:**
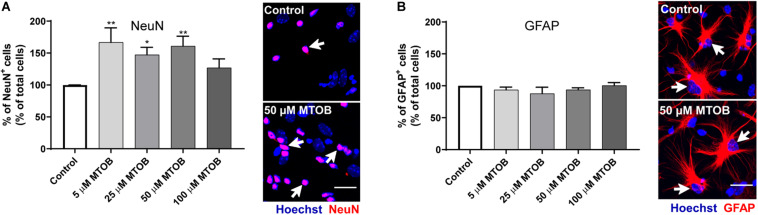
CtBP modulation promotes the differentiation of progenitor cells into neurons. **(A,B)** Bar graph depicts the percentage of **(A)** NeuN-immunostained mature neurons and **(B)** GFAP-immunostained astrocytes, maintained in culture for 7 days after MTOB treatments. Data are expressed as percentage of control ± SEM. **(A)** *n* = 5–7 and **(B)** *n* = 3 independent experiments. Control was set to 100%. *p < 0.05 and **p < 0.01 when compared with control, using one-way ANOVA, followed by Dunnett’s multiple comparison test. Representative confocal images of NeuN and GFAP immunostainings are shown next to the graphs, with white arrows highlighting staining. Nuclei are stained with Hoechst. Scale bar: 20 μm.

Next, the effects of MTOB on initial steps of SVZ cell commitment were investigated by performing co-stainings against Ki67 and different neural phenotypes, 48 h after cell treatments. MTOB did not affect the number of total Ki67^+^ cells (Figure 5A) nor the number of proliferative astrocyte-like cells (Ki67^+^/GFAP^+^), Nestin^+^ (Ki67^+^/Nestin^+^), and Olig2^+^ cells (Ki67^+^/Olig2^+^) compared to control (Figures 5B–D, respectively). Nevertheless, 25 μM of MTOB did significantly increase the number of proliferative neuroblasts in the cultures (Ki67^+^/DCX^+^) in about 1.5-fold increase when compared to control (Figure 5E; mean absolute value in control: 25%; *p* = 0.0115). Although not significant, 5 and 50 μM of MTOB also show a tendency to increase Ki67^+^/DCX^+^ cells. Considering these results, the effects of MTOB on neuronal differentiation were assessed 7 days after cell treatments. As shown in Figure 6A, 5 and 50 μM of MTOB significantly increased the percentage of mature neurons approximately in 1.7-fold increase when compared to control whereas 25 μM of MTOB significantly increased it in about 1.5-fold change (NeuN^+^; mean absolute value in control: 15%; 5 μM: *p* = 0.0071; 25 μM: *p* = 0.0433; 50 μM: *p* = 0.0098). However, no significant changes were observed in the percentage of GFAP^+^ cells (Figure 6B) when compared to control, indicating that CtBP activation promotes neurogenesis in the SVZ. Considering these results, the concentrations up to 50 μM of MTOB were selected for the following experiments.

### CtBP Modulation Induces Oligodendroglial Differentiation

**FIGURE 7:**
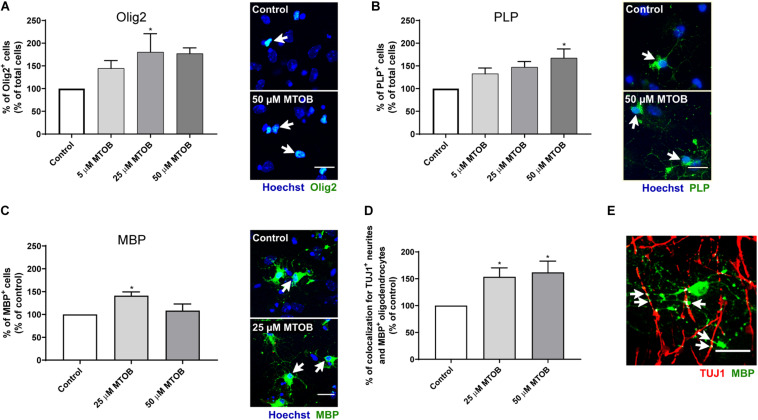
CtBP modulation induces the differentiation of progenitor cells into oligodendrocytes and enhances neurites myelination by mature oligodendrocytes. **(A–C)** Bar graphs depict the percentage of **(A)** Olig2-immunostained oligodendrocytes, **(B)** PLP-immunostained mature oligodendrocytes, and **(C)** MBP-immunostained mature oligodendrocytes, maintained in culture for 7 days after MTOB treatments. Data are expressed as a percentage of control ± SEM. **(A)** *n* = 3–4, **(B)** *n* = 4, and **(C)** *n* = 3–4 independent experiments. Control was set to 100%. *p < 0.05 when compared with control, using one-way ANOVA, followed by Dunnett’s multiple comparison test. Representative confocal images of Olig2, PLP and MBP immunostainings are shown next to the graph, with white arrows highlighting staining. Nuclei are stained with Hoechst. Scale bar: 20 μm. **(D)** Bar graph depicts the percentage of TUJ1^+^-neurites that were ensheathed by myelin sheets of MBP-mature oligodendrocytes. Data are expressed as a percentage of control ± SEM. *n* = 3–4 independent experiments. Control was set to 100%. **(E)** Representative confocal image of the visualization of myelin sheaths that enwrap neurites, with white arrows highlighting the overlay regions. Scale bar: 20 μm.

The ability of MTOB to promote oligodendroglial differentiation was also evaluated 7 days after cell treatments. Our analysis showed that 25 and/or 50 μM of MTOB increased the number of Olig2^+^ (Figure 7A; mean absolute value in control: 10%; 25 μM: 1.8-fold-increase, *p* = 0.0492), PLP^+^ (Figure 7B; mean absolute value in control: 4%; 50 μM: 1.7-fold increase, *p* = 0.0107), and MBP^+^ cells (Figure 7C; mean absolute value in control: 5%; 25 μM: 1.4-fold increase, *p* = 0.0130). These results demonstrate that the activation of CtBPs induce oligodendrogenesis in the SVZ, by increasing the number of oligodendrocyte progenitors (Olig2^+^ cells) and mature oligodendrocytes (PLP^+^ and MBP^+^ cells). Then, we investigated whether mature oligodendrocytes could myelinate the axons by performing co-stainings against MBP and TUJ1. The quantitative analysis of the intensity of colocalized pixels in TUJ1^+^ and MBP^+^ co-staining (Figure 7E) showed that 25 and 50 μM of MTOB increased the number of TUJ1^+^-neurites segments ensheathed by MBP^+^-oligodendrocytes, in about 1.5-fold increase and 1.6-fold increase, respectively, when compared to control (Figure 7D; 25 μM: *p* = 0.0424; 50 μM: *p* = 0.0317), suggesting that MTOB may also facilitate the axonal conduction of action potentials in newborn neurons. Hence, considering the effects of MTOB on neuronal and oligodendroglial differentiation, only the concentration of 25 μM of MTOB was chosen for the remaining experiment.

### CtBP Modulation Affects Neuronal Complexity

**FIGURE 8:**
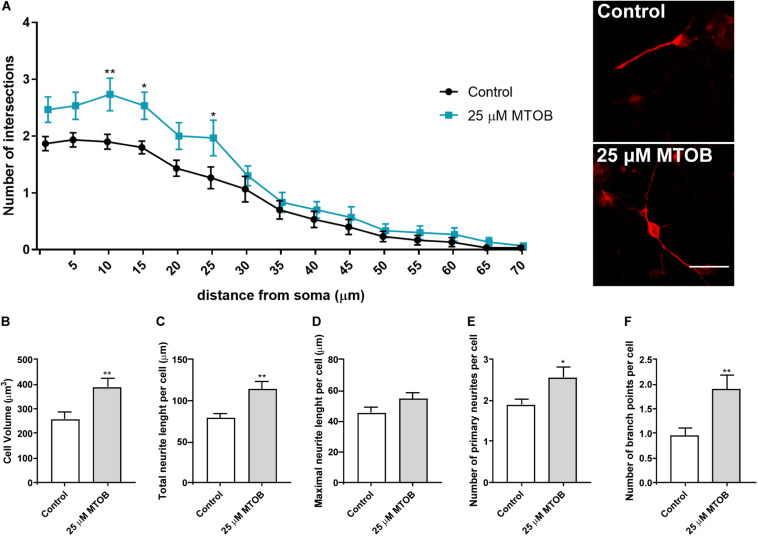
CtBP modulation increases the dendritic structure and complexity of immature neurons. **(A)** Sholl analysis of DCX^+^ cells revealed differences at several distances from the soma in the number of intersections of DCX^+^ immature neurons, after the treatment with 25 μM of MTOB for 7 days. *n* = 30 cells from three independent experiments. *p < 0.05 and **p < 0.01 when compared with control, using two-way ANOVA, followed by the Sidak’s multiple comparison test. **(B–F)** Bar graphs depict **(B)** cell volume, **(C)** total length, **(D)** maximal length, **(E)** number of primary of neurites per cell, **(F)** and the number of ramifications of neurites per cell. Data are expressed as mean ± SEM. *n* = 30 cells. *p < 0.05 and **p < 0.01 when compared with control, using unpaired Student’s *t*-test. Representative confocal images of DCX^+^ cells are shown next to the graph of Sholl analysis. Scale bar: 20 μm.

Dendritic arbor morphology and complexity are essential to determine neuronal activity and function (O’Neill et al., 2015). To investigate whether CtBPs had an impact on dendritic morphology, different parameters of dendritic structure and complexity of DCX^+^ immature neurons were analyzed at 48 h and 7 days after cell treatments with 25 μM of MTOB. It was observed that 48 h after cell treatments, MTOB did not affect the branching complexity of DCX^+^ cells measured by Sholl analysis (Supplementary Figure 3A) neither the volume, the total and maximal length nor the number of primary neurite or branching points per cell (Supplementary Figures 3B–F, respectively). Nevertheless, 7 days after cell treatment, DCX^+^ cells showed an increase in the number of dendritic intersections at several distances from the soma (Figure 8A). At this phase of maturation, DCX^+^ cells also exhibited increased volume [Figure 8B; control: 259.5 ± 25.8; 25 μM: 391.2 ± 31.9 (*p* = 0.0022)], total neurite length [Figure 8C; control: 78.4 ± 6.2; 25 μM: 113.8 ± 9.3 (*p* = 0.0024)], and number of primary neurites [Figure 8E; control: 1.9 ± 0.1; 25 μM: 2.6 ± 0.2 (*p* = 0.0170)] and ramifications per cell [Figure 8F; control: 1.0 ± 0.2; 25 μM: 1.9 ± 0.3 (*p* = 0.0072)] showing an increased maturation when compared to control. No change was seen regarding the maximal neurite length per cell (Figure 8D).

## Discussion

Neural stem cells exhibit unique characteristics, such as the ability for self-renewal and to differentiate into neurons and glial cells, revealing a tremendous potential for novel therapies regarding brain repair. At gene expression level, CtBPs are presented as novel transcriptional regulators of neurogenesis during embryonic development, unveiling their impact on the behavior of neural stem and progenitor cells from self-renewal to neuronal maturation (Xie et al., 2011; Dias et al., 2014; Karaca et al., 2020). Nevertheless, studies focusing on the role of CtBPs in the adult brain are limited. Herein, we report for the first time the expression of both CtBP1 and 2 in the SVZ niche and its effect on neuronal and oligodendroglial differentiation from NSCs.

CtBP1 and CtBP2 are expressed in most regions of the brain, in nuclear and cytoplasmic localization, from development to adulthood. In the mouse embryo, *in situ* hybridization studies demonstrated that both proteins had a particularly strong expression in the nervous system (Furusawa et al., 1999). Both CtBPs are expressed in the adult brain, including in the hippocampus, one of the main neurogenic niches. Nuclear CtBP1 was strongly detected in the CA1 region but weakly detected in the CA3 region. Interestingly, the co-localization of CtBP1 with Bassoon, a presynaptic protein, in the CA1 and the CA3 regions confirmed its localization in hippocampal synapses. CtBP2 shows a strong nuclear localization in CA3 and especially in CA2 regions, except in CA1, where no immunoreactivity was detectable. Surprisingly, CtBP2 longer isoforms showed to have a synaptic localization in the neuropil layers (Hübler et al., 2012). Regarding the expression of CtBPs in NSCs, the available information is limited and focused on embryonic development. A few studies demonstrate that CtBP1 and CtBP2 are present only in the nucleus of embryonic neural stem and progenitor cells (Dias et al., 2014; Karaca et al., 2020). In the SVZ cultured cells, we observed that both CtBPs are expressed in the nucleus of immature/progenitor Ki67^+^, Sox2^+^, Nestin^+^/GFAP^+^, and Nestin^+^/GFAP^–^. Both CtBPs are also found in SVZ-derived neurons (DCX^+^, NeuN^+^, and MAP2^+^), astrocytes (GFAP^+^, Nestin^–^/GFAP^+^) and oligodendrocytes (Olig2^+^, MBP^+^, and PLP^+^). Additionally, both CtBPs showed a similar expression at short (48 h) and long-term (7 days) protocols of differentiation, being expressed in more than 90% of the cells. However, in the SVZ neurogenic niche *in vivo*, CtBPs expression and intracellular localization present some differences. CtBP1 and CtBP2 were expressed in the nuclei of subpopulations of SVZ immature/progenitor and glial cells and in some striatal neurons. The nuclear expression observed in SVZ neural stem and progenitor cells is in line with studies reporting the nuclear CtBPs localization in cancer stem cells (Dcona et al., 2017), which share some characteristics with NSC, including self-renewal and multipotency (Rich, 2008). Moreover, the nuclear localization of CtBP1 and CtBP2 in the SVZ cells indicates that both CtBPs are acting as either transcriptional coactivators or corepressors. Interestingly, our data revealed that CtBP1 is also present in the cytoplasmic compartment of DCX^+^ cells in the SVZ neurogenic niche *in vivo*. Concomitantly, CtBP1 was shown to be highly expressed in the presynaptic compartment of neurons, where it is involved in the recycling of the synaptic vesicles (Ivanova et al., 2015, 2020). CtBP1 synapto-nuclear localization is controlled by the neuronal activity and once in the nucleus, it can control the expression of genes involved in neuroplasticity (Ivanova et al., 2015). Future studies should investigate whether CtBP1 may also modulate the synaptic transmission in the newborn neurons from the SVZ. Some differences in terms of CtBPs expression were found in *in vitro* versus *in vivo* conditions. This may be due to differences in terms of the cellular and molecular neurogenic niche found in these distinct contexts. Nonetheless, co-localization between CtBPs and the main cell phenotypes analyzed (immature cells, progenitors, neurons, astrocytes, and oligodendrocytes) was found in both systems.

To investigate the function of CtBPs in NSCs, we have used a dual modulator of CtBPs activity, displaying a high affinity for these proteins, MTOB (Achouri et al., 2007; Hilbert et al., 2014). Although MTOB has never been tested in SVZ cells, millimolar concentrations of this compound were proven to be toxic to cultured cerebellar granule neurons and several types of cancer cells, leading to apoptosis (Warner et al., 2013; Dcona et al., 2017). Our results support these data as high concentrations of MTOB (1 and 2.5 mM) induced cell death in SVZ cells. Still, the lower concentrations of MTOB used in this work were not able to protect against the basal death occurring in these cultures.

It has been reported that CtBPs regulate genes involved in cell proliferation and cancer stem cell self-renewal (Chinnadurai, 2009). In the human neuroblastoma cell line SHSY5Y and prostate cancer PC3 cells, the downregulation of CtBP2 inhibited cell proliferation and arrested the cell cycle, by decreasing the expression of c-Myc (Zhang et al., 2014; Nan et al., 2017). Furthermore, in human embryonic stem cells, the inhibition of CtBP function with MTOB significantly decreased the expression of essential transcriptional factors involved in the maintenance of self-renewal, including Sox2 (Arthur et al., 2019). Herein, we evaluated CtBPs influence in the proliferation of SVZ cells. Our results showed that total cell proliferation, evaluated by the Ki67 staining, was not affected by low concentrations of MTOB in SVZ. Furthermore, MTOB did not affect the commitment of the immature and glial population at early stages. Nevertheless, one should consider that Olig2 is a marker for both type C cells and oligodendrocytes (Mitew et al., 2014), and therefore, at this stage we could not assess CtBPs influence over oligodendroglial commitment. Interestingly, we found that 25 μM MTOB increased the number of Ki67^+^/DCX^+^. These results suggest that CtBP activation is influencing the initial steps of cell commitment without affecting the total proliferation of NSCs of the SVZ.

Considering the above results, we assessed the effects of CtBPs in neuronal and glial long-term differentiation. Low concentrations of MTOB prompted neuronal differentiation not only by increasing the number of Ki67^+^/DCX^+^ cells but also the number of NeuN-mature neurons. Furthermore, treatment with 25 μM MTOB promoted neuronal maturation of DCX^+^ cells. DCX^+^ cells presented a higher number of dendritic intersections at several distances from the soma. Also, they showed an increased volume and neurite length and a higher number of primary neurites and branch points *per* cell, suggesting that CtBPs are essential to reinforce a proper neuronal differentiation. DCX is essential for dendritic extension, growth cone formation, neuronal differentiation, maturation and migration, by virtue of its involvement in microtubule stabilization. Thus, we can also extrapolate about the role of CtBPs on other steps of neuronal differentiation and maturation, which should be investigated in future studies. In fact, CtBP2 revealed to be essential for proper neuronal differentiation of neural stem and progenitor cells during mouse developing cortex (Karaca et al., 2020). Furthermore, in the neural tube, the presence of high oxygen levels required for the expression of Hes1 leads CtBPs to be less recruited to the promoter of *Hes1*, repressing the differentiation of NSCs into neurons through BMP signaling (Dias et al., 2014). Also, Smad6, an inhibitor of the BMP pathway, was shown to promote neuronal differentiation of neural progenitor cells not only by inhibiting BMP signaling but also by enhancing the interaction of CtBPs with TCF, inhibiting Wnt/β-catenin pathway (Xie et al., 2011). In addition to neurogenesis, we also observed that CtBPs are involved in oligodendroglial differentiation as we observed an increase in the number of Olig2^+^ cells, MBP^+^ cells, and PLP^+^ cells. Olig2 is expressed at all stages of the oligodendrocyte lineage, inclusively in myelination (Mitew et al., 2014). As they become fully mature oligodendrocytes, immature oligodendrocytes start to express myelin-specific proteins including MBP and PLP, giving rise to mature myelin-producing oligodendrocytes (Boulanger and Messier, 2014). However, MBP is expressed earlier than PLP (Duchala et al., 1995), which can disclose the differences that we found regarding the effects of MTOB on Olig2^+^, MBP^+^, and PLP^+^ cells. Our data suggested that this increased number of oligodendrocytes influenced the process of neurite myelination. Altogether, our results suggest that oligodendrocytes are supporting the newly formed neurons, contributing to neural plasticity. Interestingly, treatment with low concentrations of MTOB prompted neuronal and oligodendroglial differentiation of NSCs without affecting the expression of both CtBPs, namely in NeuN^+^ and Olig2^+^ cells (data not shown). As previously mentioned, the balance between self-renewal and differentiation of NSCs is achieved by several mechanisms of regulation. Nevertheless, only a few are involved both in neuronal and oligodendroglial differentiation of NSCs (Faigle and Song, 2013). For instance, Notch activation promotes self-renewal, proliferation, and survival of NSCs as well as the migration and arborization of newborn neurons but inhibits neuronal and oligodendroglial differentiation and their ability to myelinate neurons (Ables et al., 2011; Mitew et al., 2014). In our work, CtBPs may be leading to an earlier inhibition of Notch signaling by interfering on cell commitment and directing NSCs to differentiate into myelinating oligodendrocytes and fully mature neurons. Besides, CtBPs may be coactivating other genes in the final steps of NSCs differentiation, including brain-derived neurotrophic factor (BDNF) release by astrocytes, which is involved not only in neuronal differentiation and dendritic arborization but also in oligodendrocyte differentiation and myelination (Faigle and Song, 2013; Mitew et al., 2014). Altogether, our data suggest that CtBPs may be acting in a gene-specific manner at different steps of NSCs development. Nevertheless, further molecular studies are needed to analyze into thoroughly the function of CtBPs in NSCs.

## Conclusion

In conclusion, we report that CtBP1 and CtBP2 are expressed in the SVZ niche and its activation promotes neuronal and oligodendroglial differentiation from SVZ NSCs. The numerous transcriptional mechanisms implied at the different steps of NSCs development compromise the evaluation of their specific contribution to the behavior of NSCs. Nevertheless, our data support that the modulation of CtBPs in the adult neurogenic niches in the context of brain regeneration, particularly, in both neurodegenerative and demyelinating diseases, may boost brain repair and regeneration.

## Data Availability Statement

The original contributions presented in the study are included in the article/Supplementary Materials, further inquiries can be directed to the corresponding author/s.

## Ethics Statement

The animal studies were carried out in certified facilities by qualified personnel (FELASA), according to the European and Portuguese National Authority for Animal Health directives (Direcção-Geral de Alimentação e Veterinária), and was authorized by the CICS-UBI Animal-Welfare Body and DGAV (REF: 0421/000/000/2019).

## Author Contributions

CS-A performed the experiments, collected, assembled, and analyzed the data, and wrote the manuscript. CS, ME, and TS collected the data. CS, ME, TS, ACC, and RF gave technical and scientific support to the experiments. ACC, RF, and LB provided resources needed for the completion of the work. LB designed the study, supervised the work, analyzed the data, and wrote the manuscript. All authors reviewed and approved the final manuscript.

## Conflict of Interest

The authors declare that the research was conducted in the absence of any commercial or financial relationships that could be construed as a potential conflict of interest.
