# Supplementary material for: C-Terminal Binding Proteins Promote Neurogenesis and Oligodendrogenesis in the Subventricular Zone
Source: Front Cell Dev Biol. 2021 Jan 6;8:584220. doi: 10.3389/fcell.2020.584220 (PMC7815648; doi:10.3389/fcell.2020.584220)
Supplement: Supplementary file 1 [file Data_Sheet_1.docx]

Supplementary Material

# Supplementary Figures and Tables

## Supplementary Figures


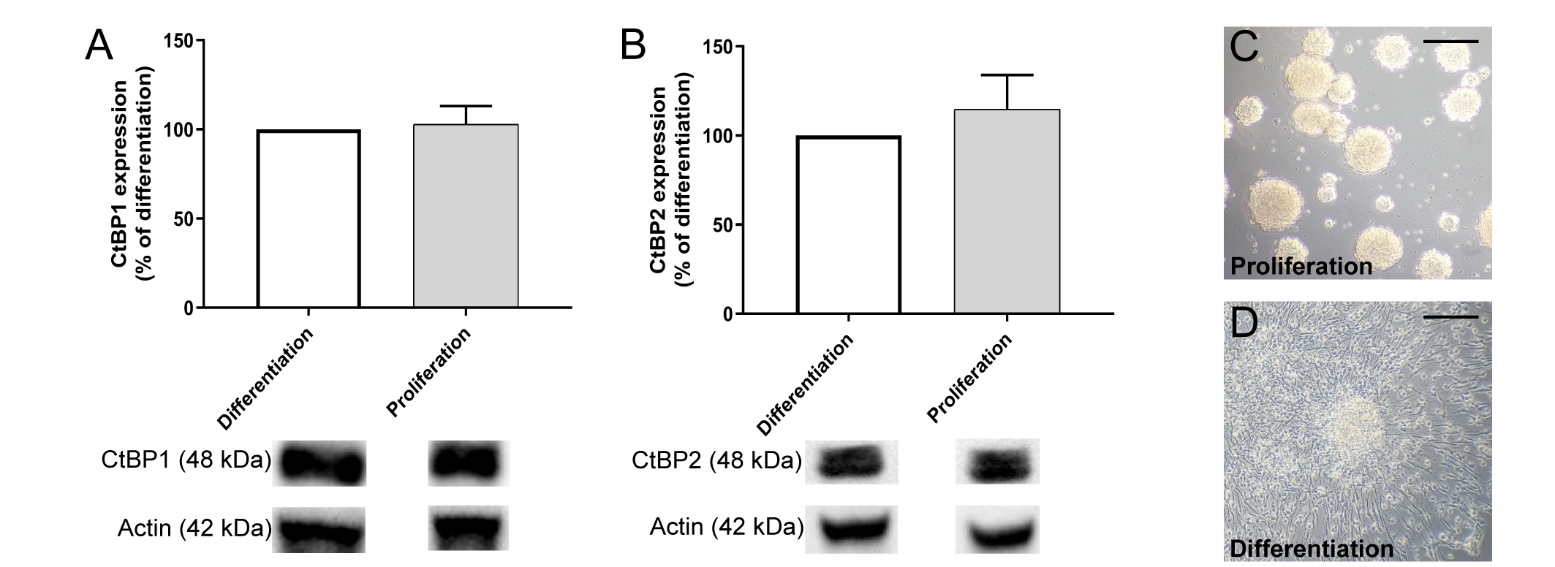


**Supplementary Figure 1.** Expression levels of CtBP1 and CtBP2 in the SVZ, *in vitro*. **(A,B)** The expression of CtBPs under proliferation conditions was evaluated in 6-day-old neurospheres whereas their expression under differentiation conditions was assessed 48 hours after 6-day-old neurospheres have been seeded in coverslips. Bar graphs depict the percentage of protein expression levels of **(A)** CtBP1 and **(B)** CtBP2 in SVZ cells, under proliferation and differentiation conditions. Protein expression was normalized to actin. Bellow each graph, representative immunoblots of CtBP1 (48 kDa), CtBP2 (48 kDa) and actin (42 kDa) are shown. Data are expressed as a percentage of differentiation ± SEM. **(A)** n = 6 and **(B)** n = 4 independent experiments. Protein expression in differentiation conditions was normalized to 100%. **(C,D)** Representative images of SVZ cells under **(C)** proliferation and **(D)** differentiation conditions. Scale bar: 100 μm.


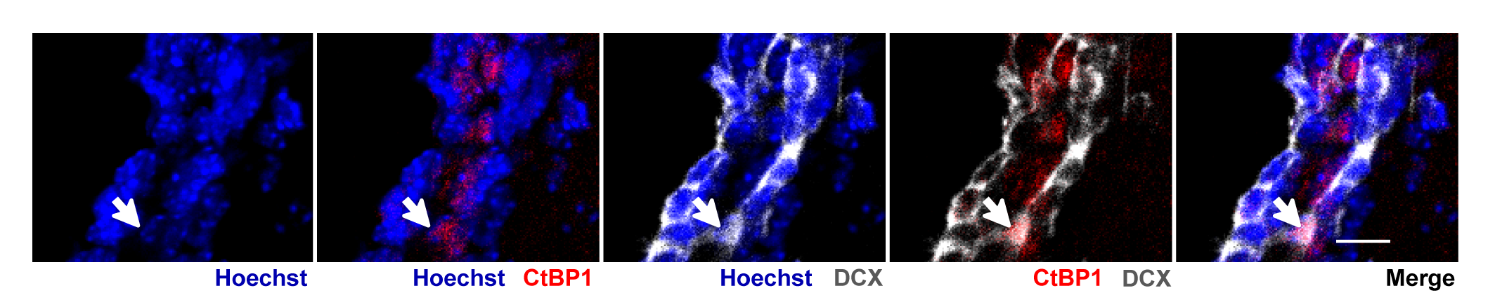


**Supplementary Figure 2.** CtBP1 presents a cytoplasmic localization in immature neurons in the SVZ neurogenic niche. Representative confocal images of the subcellular localization of CtBP1 in DCX cells evaluated in the SVZ niche of wild-type C57BL/6J adult mice. Nuclei are stained with Hoechst. White arrows highlight the DCX cell where CtBP1 is in the cytoplasmatic compartment. Scale bar: 10 μm.


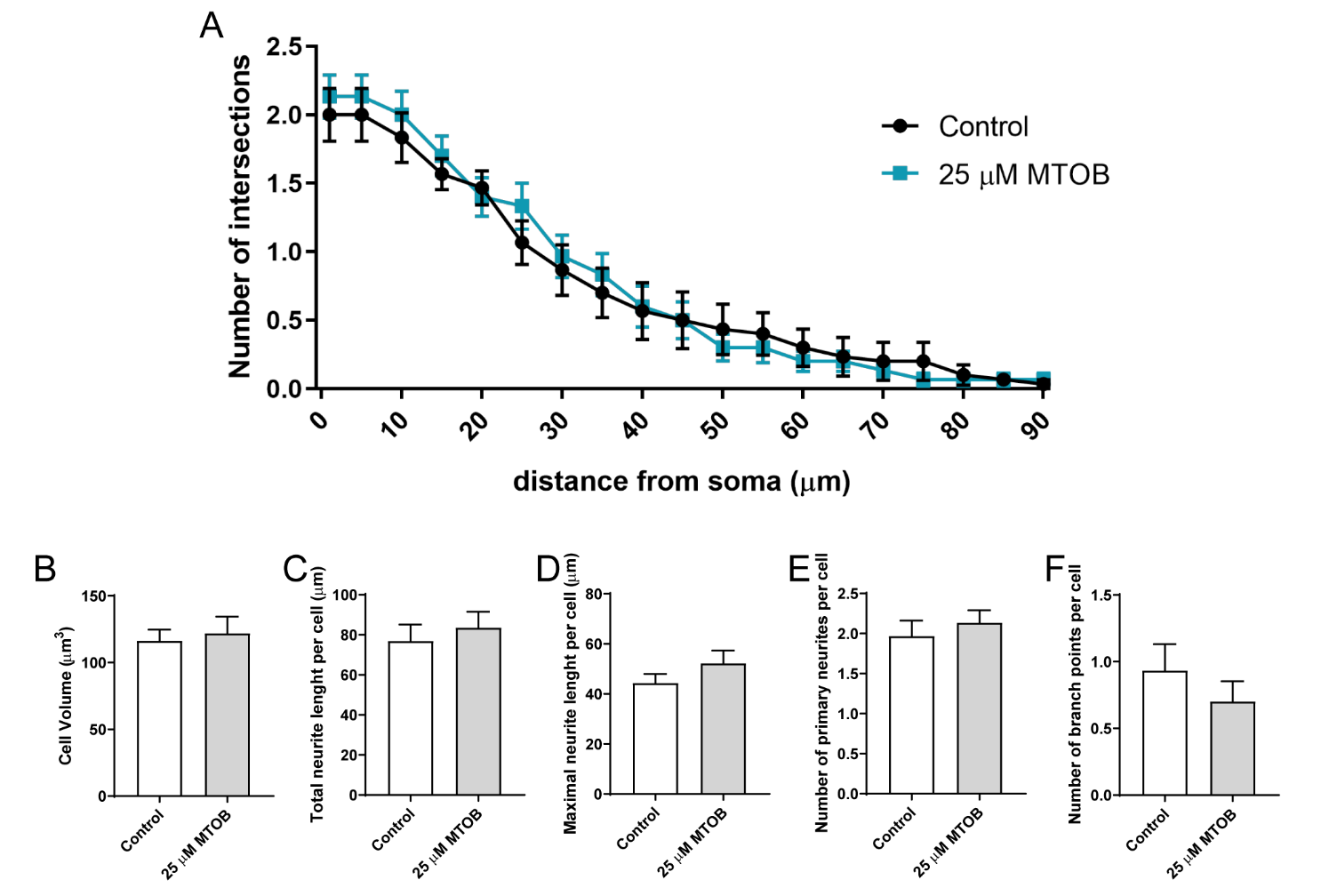


**Supplementary Figure 3.** CtBP modulation does not affect the dendritic complexity of immature neurons. **(A)** Sholl analysis of DCX^+^ cells showed no differences in the number of intersections between control and DCX^+^ immature neurons, treated with 25 μM of MTOB for 48 hours. **(B-F)** Bar graphs depict **(B)** cell volume, **(C)** total length, **(D)** maximal length, **(E)** number of primary neurites per cell **(F)** and the number of ramifications of neurites per cell. Data are expressed as mean ± SEM. n = 30 cells from 3 independent experiments.

## Supplementary Tables

**Supplementary Table 1:** Primary and secondary antibodies used for immunostainings.

| Antigen | Company | Host | Dilution ICC | Dilution IHC |
| --- | --- | --- | --- | --- |
| Primary Antibodies |  |  |  |  |
| Ki67 | Abcam Plc | Rabbit | 1:50 | 1:1000 |
| Ki67 | BD Bioscience, Franklin Lakes, NJ, U.S.A | Mouse | 1:50 | - |
| Doublecortin (DCX) | Santa Cruz Biotechnology, Inc. | Goat | 1:200 | 1:1000 |
| Nestin | Santa Cruz Biotechnology, Inc. | Goat | 1:100 | 1:200 |
| Nestin | Abcam Plc | Mouse | 1:100 | - |
| Sex-determining region Y-box 2 (Sox2) | Santa Cruz Biotechnology, Inc. | Goat | 1:200 | 1:500 |
| Glial-fibrillary acid protein (GFAP) | Dako, Denmark | Rabbit | 1:2000 | 1:2000 |
| GFAP | BD Bioscience, Franklin Lakes, NJ, U.S.A | Mouse | 1:500 | - |
| Neuronal nuclei (NeuN) | Cell Signaling Technology, Leiden, Netherlands | Rabbit | - | 1:500 |
| NeuN | Merck Millipore, Darmstadt, Germany | Mouse | 1:100 | - |
| Microtubule-associated protein 2 (MAP2) | Santa Cruz Biotechnology, Inc | Rabbit | 1:100 | - |
| Oligodendrocyte transcription factor 2 (Olig2) | Merck Millipore, Darmstadt, Germany | Rabbit | 1:200 | 1:500 |
| Proteolipid protein (PLP) | Abcam Plc | Rabbit | 1:200 | - |
| Myelin basic protein (MBP) | Cell Signaling Technology, Leiden, Netherlands | Rabbit | 1:150 | - |
| TUJ1 | BioLegend Way, San Diego, CA | Mouse | 1:600 | - |
| C-terminal Binding Protein 1 (CtBP1) | BD Bioscience, Franklin Lakes, NJ, U.S.A | Mouse | 1:200 | 1:1000 |
| C-terminal Binding Protein 2 (CtBP2) | BD Bioscience, Franklin Lakes, NJ, U.S.A | Mouse | 1:200 | 1:500 |
| Secondary Antibodies |  |  |  |  |
| Anti-Rabbit Alexa Fluor 488 | Life Technologies | Donkey | 1:200 | 1:1000 |
| Anti-Mouse Alexa Fluor 488 | Abcam Plc | Donkey | 1:200 | - |
| Anti-Goat Alexa Fluor 488 | Abcam Plc | Donkey | 1:200 |  |
| Anti-Goat Alexa Fluor 546 | Life Technologies | Donkey | 1:200 | - |
| Anti-Mouse Alexa Fluor 594 | Abcam Plc | Donkey | 1:200 | 1:1000 |
| Anti-Rabbit Alexa Fluor 594 | Abcam Plc | Donkey | 1:200 | - |
| Anti-Goat Alexa Fluor 647 | Life Technologies | Donkey | 1:200 | 1:1000 |
| Anti-Mouse Alexa Fluor 647 | Life Technologies | Donkey | 1:200 | - |

# Supplementary Material and Methods

## Western Blotting

SVZ cells cultivated under proliferation and differentiation conditions were used for western blot experiments. Proliferation condition refers to neurospheres grown in the presence of growth factors (EGF, FGF-2), which are rich in neural and progenitor stem cells capable of self-renewing and proliferating. Differentiation condition refers to neurospheres that were adhered onto PDL (0.1 mg/mL)-coated coverslips, in medium devoid of growth factors, to induce cell differentiation (Agasse et al., 2008). To obtain SVZ cells under proliferation conditions, 6-day-old neurospheres were collected from uncoated Petri dishes and were gently centrifuged at 300 rpm, for 1 minute, at RT. SVZ cells were incubated in RIPA lysis buffer (50 mM Tris, 150 mM NaCl, 1% Triton X- 100, 0.5% Sodium deoxycholate, 0.1% Sodium dodecyl sulfate (SDS), and a cocktail of protease inhibitors) at 4º C and sonicated. For differentiation conditions, 6-day-old neurospheres were allowed to adhere for 2 days onto PDL-coated 6-well plate, grown with DMEM/F-12 devoid of growth factors, in an incubator with 5% CO_2_ and 95% atmospheric air, at 37ºC. Then, SVZ cells were incubated in RIPA lysis buffer at 4º C and sonicated. The protein soluble fraction was obtained by centrifugation at 14000 rpm for 20 minutes at 4°C, and the total amount of protein was determined using the Pierce Bicinchoninic Acid Protein Assay Kit (Thermo Scientific, MA, USA).

Protein samples were denatured in loading buffer (350 mM Tris, 10% (w/v) SDS, 30% (v/v) glycerol, 9% β-mercaptoethanol and 0.06% (w/v) bromophenol blue), for 5 minutes at 95ºC. A total of 40 μg of protein was loaded into the 10% SDS polyacrylamide gels and proteins were separated by SDS-PAGE electrophoresis at 120V. Then, proteins were transferred to polyvinylidene difluoride membranes (Merck Millipore, Darmstadt, Germany), through semi-dry transfer during 25 minutes at 1.0 A, 25 V, at RT. All membranes were blocked in Tris buffer saline containing Tween 20 0.1% (TBS-T) and 0.1% gelatin (Fluka, St Louis, MO, USA), for 1 hour at RT. Thereafter, membranes were incubated overnight with mouse anti-CtBP1 (1:2500; 48 kDa; BD Bioscience, Franklin Lakes, NJ, U.S.A) and mouse anti-CtBP2 (1:2500; 48kDa; BD Bioscience, Franklin Lakes, NJ, U.S.A) antibodies, diluted in blocking solution, at 4°C. Membranes were rinsed with TBS-T and incubated the respective horseradish peroxidase-conjugated secondary antibody: goat anti-mouse (1:5000; Santa Cruz Biotechnology, Inc.), diluted in blocking solution, for 1 hour at RT. To normalize the expression of CtBPs, membranes were then incubated with a housekeeping antibody solution: mouse monoclonal anti-actin (1:5000; 42kDa; BD Bioscience, Franklin Lakes, NJ, U.S.A), followed by the respective secondary antibody, both for 1 hour at RT. Immediately before the incubation with housekeeping antibody solution, membranes were incubated with 10% hydrogen peroxide (H_2_O_2_), to inactivate the horseradish peroxidase, for 30 minutes, at RT (Danielson et al., 2018). Then, membranes were incubated with Pierce™ ECL Western Blotting Substrate (Thermo Scientific, MA, USA) for 1 minute, in the dark and protein lanes were visualized in a ChemiDoc^TM^ MP Imaging System (Bio-Rad Laboratories). Densitometric analysis was performed using the Image Lab 5.1 software (Bio-Rad Laboratories).

## Supplementary References

Danielson, A. P., Van-Kuren, D. B., Bornstein, J. P., Kozuszek, C. T., Berberich, J. A., Page, R. C., et al. (2018). Investigating the mechanism of Horseradish peroxidase as a RAFT-initiase. *Polymers (Basel).* 10, 741. doi:10.3390/polym10070741.
